# Prospective, randomized, double-blind, multi-center, Phase III clinical study on transarterial chemoembolization (TACE) combined with Sorafenib^® ^versus TACE plus placebo in patients with hepatocellular cancer before liver transplantation – HeiLivCa [ISRCTN24081794]

**DOI:** 10.1186/1471-2407-8-349

**Published:** 2008-11-26

**Authors:** K Hoffmann, H Glimm, B Radeleff, G Richter, C Heining, I Schenkel, A Zahlten-Hinguranage, P Schirrmacher, J Schmidt, MW Büchler, D Jaeger, C von Kalle, P Schemmer

**Affiliations:** 1 Department of Surgery, Ruprecht-Karls-University, Im Neuenheimer Feld 110, 69120 Heidelberg, Germany; 2 National Center for Tumor Diseases, Ruprecht-Karls-University, Im Neuenheimer Feld 350, 69120 Heidelberg, Germany; 3 German Cancer Research Center, Im Neuenheimer Feld 280, 69120 Heidelberg, Germany; 4 Department of Radiology, Ruprecht-Karls-University, Im Neuenheimer Feld 110, 69120 Heidelberg, Germany; 5 Institute of Pathology, Ruprecht-Karls-University, Im Neuenheimer Feld 220, 69120 Heidelberg, Germany

## Abstract

**Background:**

Disease progression of hepatocellular cancer (HCC) in patients eligible for liver transplantation (LTx) occurs in up to 50% of patients, resulting in withdrawal from the LTx waiting list. Transarterial chemoembolization (TACE) is used as bridging therapy with highly variable response rates. The oral multikinase inhibitor sorafenib significantly increases overall survival and time-to-progression in patients with advanced hepatocellular cancer.

**Design:**

The HeiLivCa study is a double-blinded, controlled, prospective, randomized multi-centre phase III trial. Patients in study arm A will be treated with transarterial chemoembolization plus sorafenib 400 mg bid. Patients in study arm B will be treated with transarterial chemoembolization plus placebo. A total of 208 patients with histologically confirmed hepatocellular carcinoma or HCC diagnosed according to EASL criteria will be enrolled. An interim patients' analysis will be performed after 60 events. Evaluation of time-to-progression as primary endpoint (TTP) will be performed at 120 events. Secondary endpoints are number of patients reaching LTx, disease control rates, OS, progression free survival, quality of live, toxicity and safety.

**Discussion:**

As TACE is the most widely used primary treatment of HCC before LTx and sorafenib is the only proven effective systemic treatment for advanced HCC there is a strong rational to combine both treatment modalities. This study is designed to reveal potential superiority of the combined TACE plus sorafenib treatment over TACE alone and explore a new neo-adjuvant treatment concept in HCC before LTx.

## Background

Hepatocellular carcinoma (HCC) is the sixth most common cancer worldwide with more than 1 million deaths annually [1]. The prevalence of Hepatitis B and C with consecutive liver cirrhosis and HCC tumor growth leads to an increasing incidence of HCC especially in Europe and the USA [2]. HCC develops in a cirrhotic liver in 80% of cases, with an annual incidence of 2–6% for hepatitis B virus carriers and 3–5% for hepatitis C virus infected individuals. Two decades ago, the prognosis of HCC was devastating with most patients dying within the first year after diagnosis irrespective of their treatment [3]. The development of standardized surveillance strategies and the introduction of the Barcelona-clinic liver cancer classification (BCLC) for clinical management of HCC have significantly improved outcome. In industrialized countries 30–40% of patients are now being diagnosed at initial stages when curative treatments can be optimally applied. 5-year overall survival (OS) after resection, liver transplantation (LTx) or percutaneous treatment in select candidates reaches 50% to 70% in patients with early HCC (single or 3 nodules ≤3 cm) [1]. Untreated patients at an intermediate stage (multinodular asymptomatic tumors without an invasive pattern) achieve a median OS of 16 months. Locally ablative treatments provide good results (5-year survival 40–50%) for these patients but cannot achieve response rates and outcomes comparable to surgical therapy, even when applied as a first line treatment.

LTx is the best treatment option for patients with small multinodular tumors or those with advanced liver dysfunction. It has the potential to cure the tumor and the underlying liver disease. Tumor size at the time of LTx as defined by the Milan criteria (single tumors ≤5 cm or 3 nodules ≤3 cm) has been established as a prognostic factor [4]. In tertiary referral centers applying these criteria, survival is ~70% at 5 years, with a recurrence rate of less than 15% [5]. The shortage of donors still curtails the potential benefits of LTx. In western countries, tumor progression occurs in 20–50% of cases on the transplant waiting list.

Transarterial chemoembolization (TACE) is the most widely usedneo-adjuvant treatment for HCC patients listed for LTx. Results of randomized controlled trials and meta-analyses of pooled data for patients with non-resectable HCC show a clear survival benefit after TACE compared with conservative management, and is therefore considered as standard of care in non-resectable HCC [6]. Two studies using TACE for bridging to LTx reported excellent outcomes [7,8]. Nevertheless, only patients with preserved liver function and asymptomatic multinodular tumors without vascular invasion or extrahepatic spread are eligible for TACE to avoid hepatic failure and severe adverse events [9]. Radiofrequency ablation was not considered because only few heterogenous uncontrolled studies suggested a slightly decrease in drop-out rate in patients treated with RFA before LTx and there is the risk of needle tract metastases.

Since HCC is generally considered to be chemoresistant, results of systemic therapy have previously been disappointing. Tumor response rates for single or multiple agent chemotherapy regimens were low without durable remission leading to a 1-year survival between 0% to 30% [10]. Increasing knowledge on the molecular pathogenesis of HCC has lead to the development of molecular targeted therapies. The oral multikinase inhibitor sorafenib (Nexavar^®^) blocks angiogenesis and cell proliferation in HCC. In patients with advanced HCC, sorafenib has shown a significant improvement in time-to-progression (TTP) and OS [11,12]. After the drug has now been approved by both, FDA and EMEA, it is the new reference standard treatment of patients with advanced HCC [12].

Evidence-based treatment for HCC relies on fewer than 100 randomized controlled trials and many observational studies. LTx with previous bridging by TACE is now accepted as the only treatment capable of definitely curing HCC but can only be applied to less than 30% of patients. Low response to TACE and progression on LTx waiting list are the major prognosis limitating factors. Sorafenib has direct inhibitory effects on tumor growth and maintains stable disease in many HCC patients. The combination of TACE and sorafenib represents a logical, new and encouraging approach for neo-adjuvant HCC therapy before LTx.

## Design

### Subject recruitment

The final protocol has been approved by the local ethics committee of the Ruprecht-Karls University of Heidelberg, Faculty of Medicine (EudraCT-Nr.: 2008-002269-29). The medical secrecy and the Federal Data Protection Act will be followed. Written informed consent has to be obtained. 208 patients (male or female) over 18 years of age with HCC (single nodule -8 cm or maximum of 3 nodules ≤3 cm) without extrahepatic disease potentially eligible for LTx will be enrolled in the study. Recruitment will start in October 2008. Patients are considered for recruitment to the study according to inclusion and exclusion criteria (Table 1).

**Table 1:** Criteria for inclusion and exclusion of patients

**Inclusion criteria**	**Exclusion criteria**
• Men and women >18 years of age	• Prior systemic anticancer therapy or local tumor therapy (i.e. LITT; PEI, cryotherapy, RFA, TACE)
• HCC (single nodule -8 cm or max. 3 nodules ≤3 cm) diagnosed by histology or non-invasive EASL criteria	• Significant cardiovascular disease such as myocardial infarction < 6 months previously, chronic heart failure (revised NYHA grade III-IV) or unstable coronary artery disease
• Baseline CT or MRI and bone scan without evidence of radiologically definable major vascular invasion or extrahepatic disease	• Extrahepatic disease, portal vein or other major vascular involvement;
• Hb >9.0 g/%, WBC >3.000 cells/mm^3 ^(ANC >1.500 cells/mm^3^), platelets >75.000 cells/mm^3^, bilirubin <3 mg/dl	• Uncontrolled hypertension despite optimal management
• Karnofsky index >70%	• Thrombotic or embolic events including transient ischemic attacks within the past 6 months
• Bilateral renal function with serum creatinine <1.5 mg/dl	• Hemorrhage/bleeding event = Grade 3 within 4 weeks of first dose of study drug
• INR/PTT < 1,5 × upper limit of normal	• Patients with previous malignancy other than carcinoma in situ of the skin and the cervix within the past 5 years prior to treatment
• Written informed consent	• Pregnant or breastfeeding patients.
	• Patients with uncontrolled infections or HIV seropositive patients
	• Mental conditions rendering the patient incapable to understand the nature, scope, and consequences of the study. No patient will be enrolled in this study more than once
	• Prior organ transplant (e.g. renal Tx)
	• Concomitant immunosuppressive treatment (before LTx)
	• Patients not eligible for LTx. Severe pulmonary disease that would be hazardous for LTx

### Trial coordination

The trial is coordinated by the Department of Surgery at the Ruprecht-Karls-University, Heidelberg, Germany in cooperation with the National Centre for Tumor Diseases (NCT), including overall trial management, trial registration (International Standard Randomized Controlled Trial Number (ISRCTN24081794, ), database management, quality assurance including monitoring, reporting and the scientific program of all trial related meetings.

### Data safety monitoring board

An independent data safety monitoring board (DSMB) monitors closely the proper conduct of the trial and all SAE reports to ensure the safety of the subjects during the course of the study. This committee consists of three independent physicians (2 surgeons, one oncologist) as well as a biometrician and decides on the final diagnostic classification of critical clinical events. For all serious adverse events, the documentation and relevant patient data are verified by the coordinating personnel before submitting the data to the Adverse Events Committee for diagnostic classification. Analysis of safety related data is performed with respect to frequency of:

• Serious adverse events and adverse events stratified by body-system

• Adverse events stratified by severity

• Adverse events stratified by causality.

Patient toxicities will be assessed using the NCI Common Toxicity Criteria (CTC-AE V3.0). Toxicity will be evaluated pretreatment, bi-weekly during the first month, every four weeks during the following time of treatment and at follow-up. Unacceptable toxicity is defined as unpredictable or irreversible Grade 4 toxicity. Decisions regarding sorafenib treatment and dose-adjustment will be made using the guidelines of the actual sorafenib prescribing information.

### Medication supply

The chemotherapeutic agent for TACE is prepared and provided by the pharmacy of each study center. Sorafenib and the placebo are prepared and supplied by Bayer Health Care GmbH and packaged and labeled for the trial by the pharmacy of the University Hospital Heidelberg.

### On site monitoring

The study is performed according to the principles of the ICH-GCP consolidated guidelines as required by regulatory agencies and the ethical principles according to the current revision of the Declaration of Helsinki and local legal and regulatory requirements [13,14]. The trial is monitored by KKS Heidelberg according to their standard operational procedures based on ICH-GCP guidelines.

### Study design

**Figure 1:**
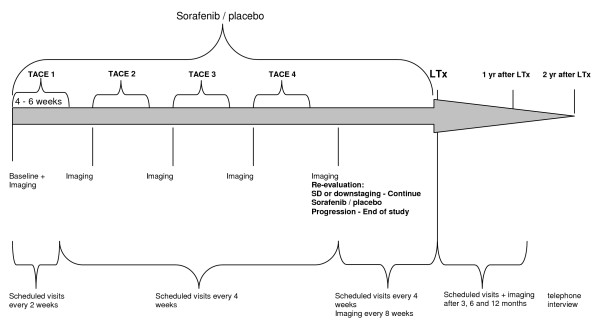
Treatment schedule.

The HeiLivCa trial is a prospective, multi-center, placebo-controlled, randomized, double-blind clinical trial with two parallel treatment groups receiving TACE plus sorafenib or TACE plus placebo. Patients in both groups will be treated with a maximum of four TACE procedures. For TACE carboplatin will be used as chemotherapeutic drug and lipiodol as embolizing agent. Four weeks after TACE, response will be evaluated by CT-imaging. Study medication will be stopped at progression. For patients without progression, treatment with sorafenib or placebo will be continued until LTx. All patients will be followed for 24 month after end of treatment. The duration of the overall trial is expected to last approximately 48 months. The actual duration of trial may vary due to the availability of patients meeting the criteria after registration for LTx. (Figure 1)

### Study objectives

The primary objective is to evaluate the efficacy of TACE plus Sorafenib compared to TACE plus placebo in patients with HCC before LTx. The primary endpoint is the TTP based on radiological assessments, defined as the time from the date of randomization to disease progression. In the absence of a TTP event, TTP time will be censored at the date of last disease assessment. The secondary efficacy objectives are to assess the number or patients reaching LTx, disease control rates, 1- and 2-year OS after LTx, progression-free survival, number of TACE in each arm, quality of life, safety and toxicity.

### Randomization and treatment

Patients will be randomly assigned on a 1:1 basis in a blinded fashion to the Sorafenib 400 mg or placebo. To accomplish this, a computer generated randomization list, which is provided to the Pharmacy at University Hospital Heidelberg, will be prepared by the Patient and Clinical Study Center (PaSZ) at NCT.

When ordering study medication, the investigator will receive a set of sealed envelopes, via the distributing pharmacy, one for each randomization number. An identical set of sealed envelopes will be held in safe and confidential custody at the NCT. These envelopes contain information on the subject's trial medication and are to be opened only under circumstances in which it is medically imperative to know what the subject is receiving. Date and reason for opening a sealed envelope must be documented. If possible, the investigator will confer with the safety officer before unblinding. The randomization envelopes are not to be opened by the investigator at the end of the trial. All envelopes will be collected by the site monitor at the end of the trial.

### Investigation schedule and follow-up

Pre-treatment evaluation for all patients includes demographic data, medical history, physical examination, co-morbidities, and concomitant medications, complete lab (CBC, hemoglobin, hematocrit, sodium, potassium, magnesium, calcium, chloride, BUN, creatinine, ASAT, ALAT, GGT, alkaline phosphatase, total bilirubin, direct/indirect bilirubin, lipase, amylase, lactat dehydrogenase, total protein, albumin, glucose, bicarbonate, autoantibodies, PTT, PT and INR, tumor marker AFP, hepatitis virology, TSH, fT3 and fT4) and radiological studies to meet the inclusion criteria. During treatment, patients will visit the outpatient clinics every two weeks during the first month and every four weeks thereafter. At this visits the aforementioned laboratory measurements, physical examination, concomitant medication, vital signs, Karnofsky status and adverse events will be recorded in the electronic case report form (eCRF). During each monthly visit, patients will be provided with the required monthly amount of study product (or placebo) and are asked to return the emptied study boxes to survey compliance. Four weeks after each TACE imaging study, a CT scan of the thorax and abdomen as well as analysis of tumor marker AFP will be performed. At end of treatment due to progression or day of LTx, the aforementioned laboratory measurements, physical examination, concomitant medication, vital signs and adverse events will be recorded, imaging studies and AFP level analysis performed. In the first year after treatment, patients will be seen every 3 months for clinical assessment, laboratory analysis and imaging studies. Follow-up data of OS will be evaluated after 12 and 24 months after end of treatment or LTx.

### Assessment of quality of life

Measurement of quality of life is one of the secondary objectives of the trial. OS, return to previous employment, persistence of symptoms, the ability to perform appropriate activities and to care for oneself are criteria applied in the three questionnaires used in this study. The EORTC QLQ-C30 is a general measure of quality of life in cancer patients. It consist of nine multi-item scales: five functional scales (physical, role, cognitive, emotional, and social); three symptom scales (fatigue, pain, and nausea and vomiting); and a global health and quality of life scale [15]. Specific symptoms (dyspnea, insomnia, anorexia, constipation, and diarrhea) are measured as single items. To assess disease-specific symptoms for patients with HCC the HCC specific module (QLQ HCC18) will be used in this study [16]. Patients will complete the quality of life questionnaires at the baseline visit, four weeks after each TACE and at the end of study at day of LTX or tumor progression. Health-related Quality of Life (HQoL) subscales and single item sub-scores will be summarized by the mean and median for each arm and plotted by time. HQoL data will be analysed using ANCOVA techniques. Detailed biometric analysis will be defined in the statistical analysis plan (SAP) which has to be authorized before unblinding by the biometrician, the sponsor, and the leading clinical investigator (LKP).

### Sample size calculation

The sample size calculation is based on the detection of significant differences in TTP, the primary endpoint parameter of this trial, assuming that median TTP is 4.5 months for the placebo-arm and 7.5 months for the sorafenib-arm [11]. A total of 120 TTP events are required for a log-rank test with an overall one-sided significance level of 0.05 and power of 0.875. Applying a 1:1 randomization, a planned accrual period of 24 months, and a follow-up period of 9 months, it is estimated that 136 patients will be needed. From the experience gained at the surgery department it can be expected that about 50% of the patients will drop-out after randomization. These patients do not contribute any information to the primary endpoint of this study. In order to accommodate for a maximum drop-out rate of 50% the total sample size is therefore increased to 208.

A design with 2 stages (one interim analysis and the final analysis) has been chosen. The critical values and the test characteristics of the group sequential test design were calculated for the O'Brien and Fleming stopping rule. The nominal significance levels for the interim analysis (planned to be conducted when 60 events have occurred) and final analysis are 0.0051 and 0.0484 respectively. The final analysis will take place when approximately 120 events are observed.

### Statistical considerations

Full analysis set will include all patients who are randomized, with study medication according to initial randomization, regardless of whether patients receive study medication or receive a different drug from that to which they were randomized. This population will be the primary population for evaluating the efficacy. Safety Population: The safety population consists of all patients who received at least one dose of study medication. This population will be the primary population for evaluating treatment administration/compliance and safety. TTP will be compared between arm A and arm B using the log-rank test (primary analysis). Median TTP with corresponding 95% confidence intervals (CI) will be estimated using Kaplan-Meier methods. The hazard ratio (arm B/arm A) will be estimated by proportional hazard regression. PFS and OS will be compared between arm A and arm B using a log-rank test stratified by the variables LTx (yes or no) and center. Median survival with corresponding 95% CIs will be estimated using Kaplan-Meier methods. The hazard ratio (arm B/arm A) will be estimated by proportional hazard regression with treatment and the factors used in the stratified log-rank test in the model. Estimates of the rates of patients reaching LTx in both arms, disease control rates, response rates, frequencies of TACE, overall survival, progression free survival and 95% confidence interval will be calculated for each treatment group. Response rates will be compared between treatment groups using the Cochran-Mantel-Haenszel test adjusting by the variable LTx (yes or no) and center. The health-related quality of life and disease/treatment-related symptoms scales will be scored according to the EORTC recommendations as described in the EORTC QLQ-C30 and QLQ-HCC18 scoring manual. Health-related quality of life subscales and single item subscores will be summarized by the mean and median for each arm and plotted by time. The change from baseline for all domains will be examined by treatment arm. All patient data (clinical and resource use) generated during the study will be recorded on the eCRFs specifically designed to meet the data recording requirements of the clinical study protocol. All data management activities will be done according to ICH-GCP guidelines. Responsibility for data management is the NCT. Throughout the study, all patient information in the eCRF will only be identifiable by means of an identification number (patient number) and patient initials.

## Discussion

Tumor progress while waiting for LTx is common in HCC patients. Drop-out rates range between 30% to 50% [17]. The role of neo-adjuvant therapies during the time on the waiting list remains a matter of debate. Currently, TACE is the standard for bridging patients and has in addition shown good results for downstaging patients initially not eligible for LTx [18-20]. This information is based on case series, case-control studies, and cohort studies showing a 22–29% rate of complete necrosis for TACE [21-25]. Two studies employing TACE for bridging to LTx reported excellent outcome [7,8]. Nevertheless, recurrence rates were only low when applying the Milan criteria [4]. The impact of recent new treatment approaches; expanding transplant criteria and the availability of a systemic therapy with sorafenib on drop-out rate, recurrence and OS have to be assessed in randomized studies.

The HeiLivCa trial is an randomized, controlled, double-blind, multi-center trial attempting to maximize tumor growth control in patients with HCC awaiting LTx by adding sorafenib the most effective currently available systemic treatment to brdging with TACE, the current standard of care. Progression-free survival is the best surrogate endpoint in most solid malignancies, but it is a vulnerable endpoint in HCC since the underlying liver disease is a comorbidity with significant mortality that would potentially bias the evaluation of efficacy in this setting. According to the recommendation of a recent consensus conference of the AASLD and the EASLD, TTP is chosen as a more valid primary end-point. With regard to the potential side effects and possible cumulative adverse events in combination with TACE and LTx safety, efficacy and quality of life will be analyzed as secondary endpoints.

## Abbreviations

AASLD: American Association for Study of Liver Diseases; AFP: α-Fetoprotein; ALAT: Alanine Amino Transferase, also known as SGPT; ASAT: Aspartate Amino Transferase, also known as SGOT; BCLC: Barcelona-clinic liver cancer bid twice a day; BUN: blood urea nitrogen; CBC: complete blood count; CTC: Common Toxicity Criteria; EASLD: European Association for Study of Liver Diseases; Ecrf: electronic case report form; EMEA : European Medicines Agency; FDA: Food and Drug Agency; GGT : Gamma Glutamyl Transpeptidase; HBV: Hepatitis B virus; HCV: Hepatitis C virus; HCC: hepatocellular carcinoma; ICH-GCP: International Conference on Harmonization of Technical Requirements for Registration of Pharmaceuticals for Human Use – Good Clinical Practice; ISRCTN: International Standard Randomized Controlled Trial Number; LTx: liver transplantation; NCI: National Cancer Institute; NCT: National Center for Tumor Diseases; PT: Prothrombin Time; PTT: Partial Thromboplastin Time; PT/INR: Prothrombin time; TACE: transarterial chemoembolization; TTP: time-to-progression.

## Competing interests

The authors declare that they have no competing interests.

## Authors' contributions

KH, PS, HG, DJ and CvK conceived and designed the study based on their preclinical and clinical results and experiences, developed essential study documents, and formulated the statistical analysis plan. KH and PS performed quality review to assure adherence to current guidelines and laws. BR, PSch and GR supported the preparation of the study protocol. JS and MWB supported the design of the study with their knowledge and experience. KH conducts the study as the principal investigator. KH and PS wrote the manuscript. IS planned the statistical analysis and AZH coordinates administrative project management. All authors read and approved the final manuscript.

This study is supported in parts by a grant from Bayer Vital GmbH, Germany.

## Pre-publication history

The pre-publication history for this paper can be accessed here:


